# The long noncoding RNA MALAT1 promotes tumor-driven angiogenesis by up-regulating pro-angiogenic gene expression

**DOI:** 10.18632/oncotarget.6675

**Published:** 2016-02-02

**Authors:** Andrew E. Tee, Bing Liu, Renhua Song, Jinyan Li, Eddy Pasquier, Belamy B. Cheung, Cizhong Jiang, Glenn M. Marshall, Michelle Haber, Murray D. Norris, Jamie I. Fletcher, Marcel E. Dinger, Tao Liu

**Affiliations:** ^1^ Children's Cancer Institute Australia for Medical Research, Randwick, NSW, Australia; ^2^ Advanced Analytics Institute, University of Technology, Sydney, Broadway, NSW, Australia; ^3^ Metronomics Global Health Initiative, Marseille, France; ^4^ Shanghai Key Laboratory of Signaling and Disease Research, School of Life Sciences and Technology, Tongji University, Shanghai, China; ^5^ Kids Cancer Centre, Sydney Children's Hospital, Randwick, NSW, Australia; ^6^ Centre for Childhood Cancer Research, University of New South Wales, Sydney, Kensington, NSW, Australia; ^7^ Garvan Institute of Medical Research, Sydney, Darlinghurst, NSW, Australia; ^8^ St Vincent's Clinical School, Faculty of Medicine, University of New South Wales, Sydney, Darlinghurst, NSW, Australia

**Keywords:** long noncoding RNA, MALAT1, angiogenesis, neuroblastoma, FGF2

## Abstract

Neuroblastoma is the most common solid tumor during early childhood. One of the key features of neuroblastoma is extensive tumor-driven angiogenesis due to hypoxia. However, the mechanism through which neuroblastoma cells drive angiogenesis is poorly understood. Here we show that the long noncoding RNA MALAT1 was upregulated in human neuroblastoma cell lines under hypoxic conditions. Conditioned media from neuroblastoma cells transfected with small interfering RNAs (siRNA) targeting MALAT1, compared with conditioned media from neuroblastoma cells transfected with control siRNAs, induced significantly less endothelial cell migration, invasion and vasculature formation. Microarray-based differential gene expression analysis showed that one of the genes most significantly down-regulated following MALAT1 suppression in human neuroblastoma cells under hypoxic conditions was fibroblast growth factor 2 (FGF2). RT-PCR and immunoblot analyses confirmed that MALAT1 suppression reduced FGF2 expression, and Enzyme-Linked Immunosorbent Assays revealed that transfection with MALAT1 siRNAs reduced FGF2 protein secretion from neuroblastoma cells. Importantly, addition of recombinant FGF2 protein to the cell culture media reversed the effects of MALAT1 siRNA on vasculature formation. Taken together, our data suggest that up-regulation of MALAT1 expression in human neuroblastoma cells under hypoxic conditions increases FGF2 expression and promotes vasculature formation, and therefore plays an important role in tumor-driven angiogenesis.

## INTRODUCTION

Neuroblastoma, originating from precursor neuroblast cells in the sympathetic nervous system, is the most common extracranial malignancy in children, and accounts for 15% of all childhood cancer deaths [1]. One of the key features of human neuroblastoma tissues is extensive tumor-driven angiogenesis [2].

Angiogenesis is a pre-requisite for tumor progression and metastasis [3]. When placed in a state of prolonged or severe reduction of tissue oxygen tension, or hypoxia, cancer cells undergo adaptive changes that allow them to up-regulate the expression of pro-angiogenic genes, leading to tumor-driven angiogenesis, tumor cell survival, proliferation and metastasis [4]. The genes most important for the tumor-driven angiogenic switch include Hypoxia-Inducible Factor 1α (HIF1α), Vascular Endothelial Growth Factor and Fibroblast Growth Factor 2 (FGF2) [5].

Long noncoding RNAs (lncRNAs) are transcripts more than 200 nucleotides in length without a functional open reading frame. According to their genetic location in relation to neighbouring protein-coding genes, lncRNAs can be divided into sense, antisense, bidirectional, intronic and intergenic subtypes [6, 7]. In the last several years, lncRNAs have emerged as essential mediators of gene transcription, mRNA stabilization/degradation, cell proliferation, survival, tumour initiation and metastasis [6, 7]. For example, the lncRNA lncUSMycN binds to the RNA-binding protein NonO and up-regulates N-Myc mRNA and protein expression [8]. The lncRNA lincRNA-p21 is directly up-regulated by the tumor suppressor p53 and plays an important role in p53-regulated apoptosis [9]. In addition, the lncRNA HOTAIR regulates the expression of genes critical for tumor cell invasion, and plays a critical role in breast cancer cell invasion and metastasis [10].

The long intergenic noncoding RNA MALAT1, also known as NEAT2, is over-expressed in metastatic, compared with primary, cancer tissues, and high levels of MALAT1 expression in cancer tissues correlate with poor patient prognosis [11–13]. Recent studies have shown that MALAT1 suppression impairs cancer cell migration, invasion and metastasis, suggesting an important role of MALAT1 in cancer metastasis [12, 14, 15].

We have previously shown that MALAT1 gene expression is up-regulated by the histone demethylase JMJD1A in neuroblastoma cells, and that MALAT1 induces neuroblastoma cell migration and invasion [16]. In this study, we examined the role of MALAT1 in tumor-driven angiogenesis. Our results showed that up-regulation of MALAT1 in neuroblastoma cells as a result of hypoxia, induced endothelial cell migration, invasion and vasculature formation. The mechanism of action mediating these effects involved up-regulation of the pro-angiogenic factor, FGF2.

## RESULTS

### Hypoxia increases MALAT1 gene expression but MALAT1 does not affect cell proliferation in neuroblastoma cells

**Figure 1:**
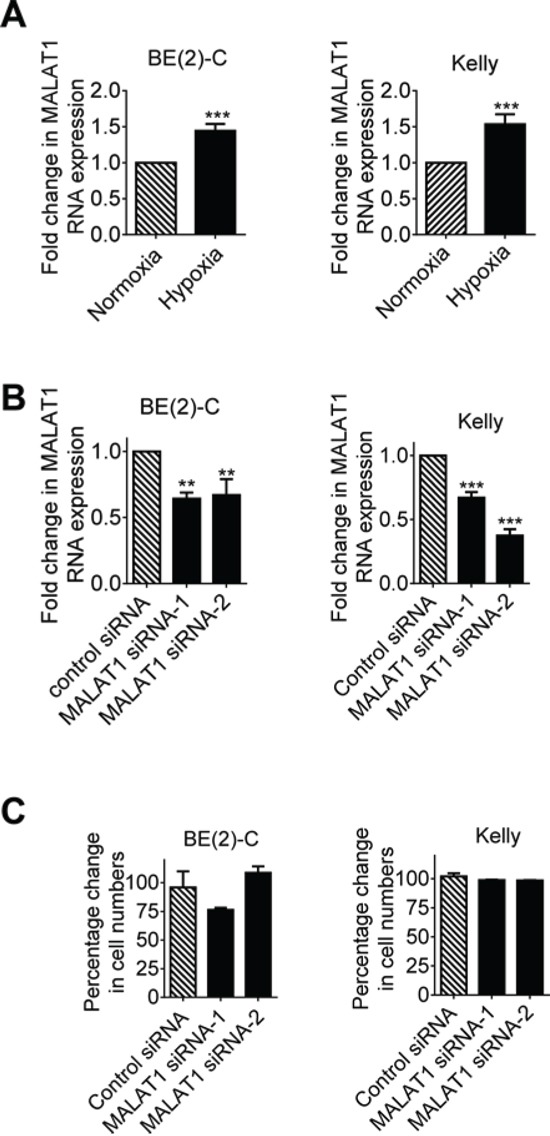
Hypoxia increases MALAT1 gene expression but MALAT1 does not affect cell proliferation in neuroblastoma cells **A.** BE(2)-C and Kelly neuroblastoma cells were cultured under normoxic or hypoxic conditions for 48 hours, followed by RT-PCR analysis of MALAT1 gene expression. **B.** BE(2)-C and Kelly cells were transfected with control siRNA, MALAT1 siRNA-1 or MALAT1 siRNA-2 for 48 hours under hypoxic conditions, followed by RT-PCR analysis of MALAT1 gene expression. **C.** BE(2)-C and Kelly cells were transfected with control siRNA, MALAT1 siRNA-1 or MALAT1 siRNA-2 for 72 hours under hypoxic conditions, followed by Alamar blue assays to quantitate viable cell numbers. Relative numbers of cells were expressed as a percentage change compared with samples transfected with control siRNA. Error bars represented standard error. ** and *** indicated *P* < 0.01 and 0.001 respectively.

Since MALAT1 is well-known to be over-expressed in metastatic, compared with primary, human tumor tissues [11, 12, 17], and hypoxia is known to induce tumour cell metastasis, we examined whether hypoxia up-regulated MALAT1 gene expression in human neuroblastoma cell lines. As shown in Figure 1A, MALAT1 gene expression was increased in human neuroblastoma BE(2)-C and Kelly cells cultured under hypoxic conditions (1% O_2_) for 48 hours, by comparison with cells cultured under normoxic conditions. To determine whether MALAT1 plays a role in neuroblastoma cell proliferation, we transfected BE(2)-C and Kelly cells with control siRNA or independent MALAT1 siRNAs (MALAT1 siRNA-1 or MALAT1 siRNA-2) under hypoxic conditions, followed by RT-PCR analysis of MALAT1 gene expression and Alamar blue assays of the numbers of cells. RT-PCR analysis confirmed that MALAT1 siRNAs reduced MALAT1 gene expression (Figure 1B), but Alamar blue assays showed that knocking-down MALAT1 expression did not have an effect on the numbers of neuroblastoma cells (Figure 1C). The data suggest that MALAT1 gene expression is up-regulated by hypoxia but MALAT1 does not modulate neuroblastoma cell proliferation under hypoxic conditions.

### MALAT1 expression in neuroblastoma cells induces endothelial cell migration, invasion and vasculature formation

**Figure 2:**
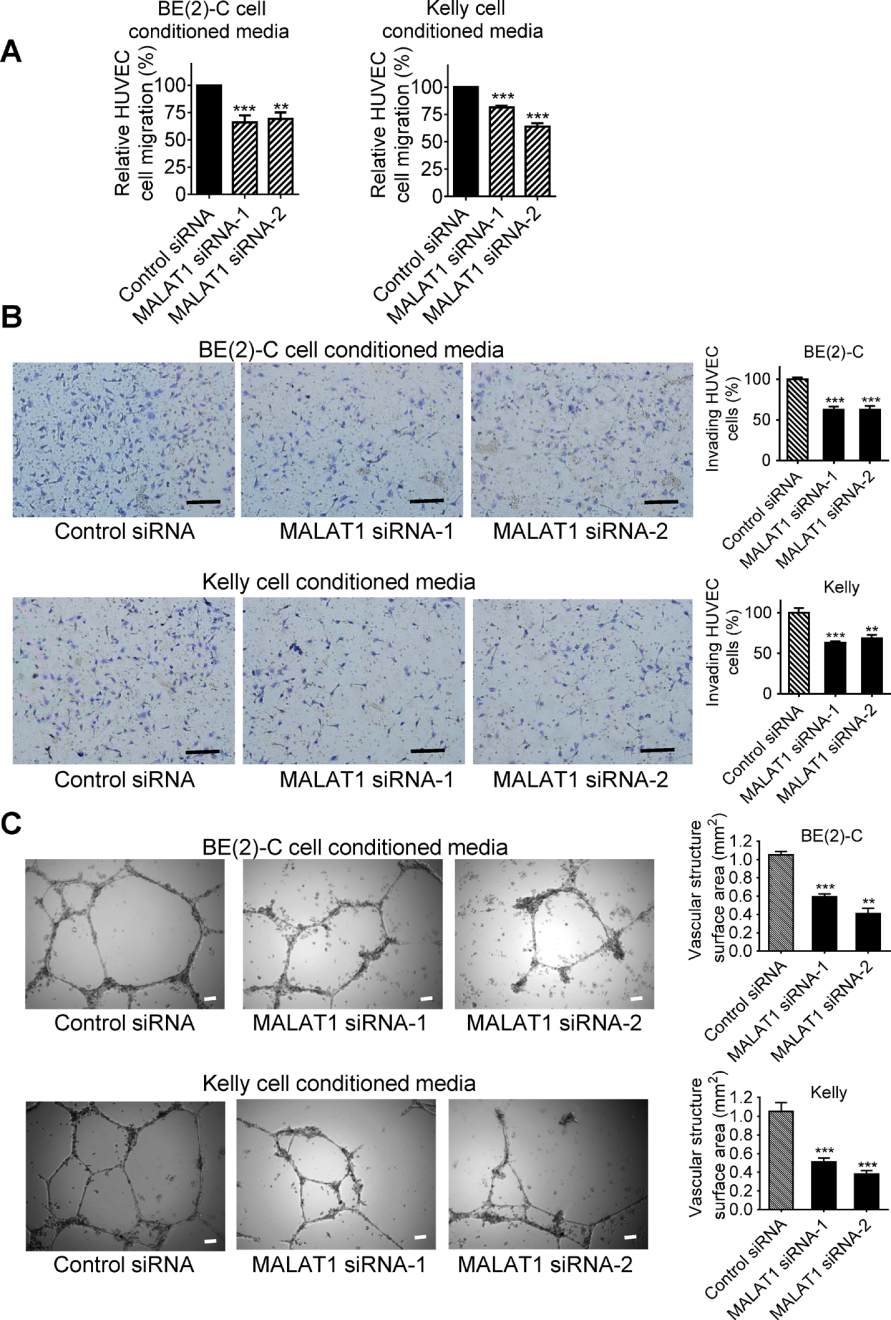
MALAT1 expression in neuroblastoma cells induces endothelial cell migration, invasion and vasculature formation BE(2)-C and Kelly neuroblastoma cells were transfected with control siRNA, MALAT1 siRNA-1 or MALAT1 siRNA-2 for 72 hours under hypoxic conditions, and cell culture media were collected. **A.** For HUVEC cell migration assays, the conditioned cell culture media were added into fluorescently labeled HUVEC cells in the upper part of chemotaxis chambers. HUVEC cells were allowed to migrate through 8-μm pore polyethylene terephthalate membrane towards chemoattractants in the lower part of the chemotaxis chambers for 6 hours. The lower part of the chemotaxis chamber was read with a fluorescence plate reader at 492/517 nm, and the relative numbers of HUVEC cells were calculated. **B.** For HUVEC cell invasion assays, the neuroblastoma cell conditioned media were added into the lower part of cell invasion chambers. HUVEC cells were plated into the upper part of the invasion chambers and allowed to invade through membranes coated with matrigel towards the conditioned cell culture media overnight for 18 hours at 37°C. Cells invaded to the other side of the membrane were then fixed, stained, visualized under a microscope and quantified. **C.** For vasculature formation assays, HUVEC cells were cultured in matrigel-coated 24-well plates and the neuroblastoma cell conditioned media were added to the HUVEC cells for 8 hours at 37°C. Photographs of vascular structures were taken using a 5 × objective. Vasculature formation was evaluated by measuring the total surface area of capillary tubes formed in at least 10 randomly selected fields per well. Scale bars represented 100 μm. Error bars represented standard error. ** and *** indicated *p* < 0.01 and 0.001 respectively.

We have previously shown that up-regulation of MALAT1 gene expression induces neuroblastoma cell migration and invasion [16]. We next examined whether knocking-down endogenous MALAT1 expression in neuroblastoma cells under hypoxic conditions modulated endothelial cell migration, invasion and vasculature formation. BE(2)-C and Kelly neuroblastoma cells were transfected with control siRNA, MALAT1 siRNA-1 or MALAT1 siRNA-2 under hypoxic conditions for 72 hours. Conditioned cell culture media were collected and added to HUVEC cells for HUVEC cell trans-well migration, trans-matrigel invasion and vasculature formation assays. As shown in Figure 2A, HUVEC cell migration was significantly reduced when stimulated with conditioned media from BE(2)-C and Kelly cells transfected with MALAT1 siRNAs, compared with control siRNA. Likewise, HUVEC cell invasion through matrigel and vasculature formation were both significantly reduced when stimulated with conditioned media from BE(2)-C and Kelly cells transfected with MALAT1 siRNAs, compared with control siRNA (Figures 2B, 2C). Taken together, the data suggest that up-regulation of MALAT1 in neuroblastoma cells under hypoxic conditions stimulates endothelial cell migration, invasion and vasculature formation.

### MALAT1 expression in endothelial cells induces vasculature formation

**Figure 3:**
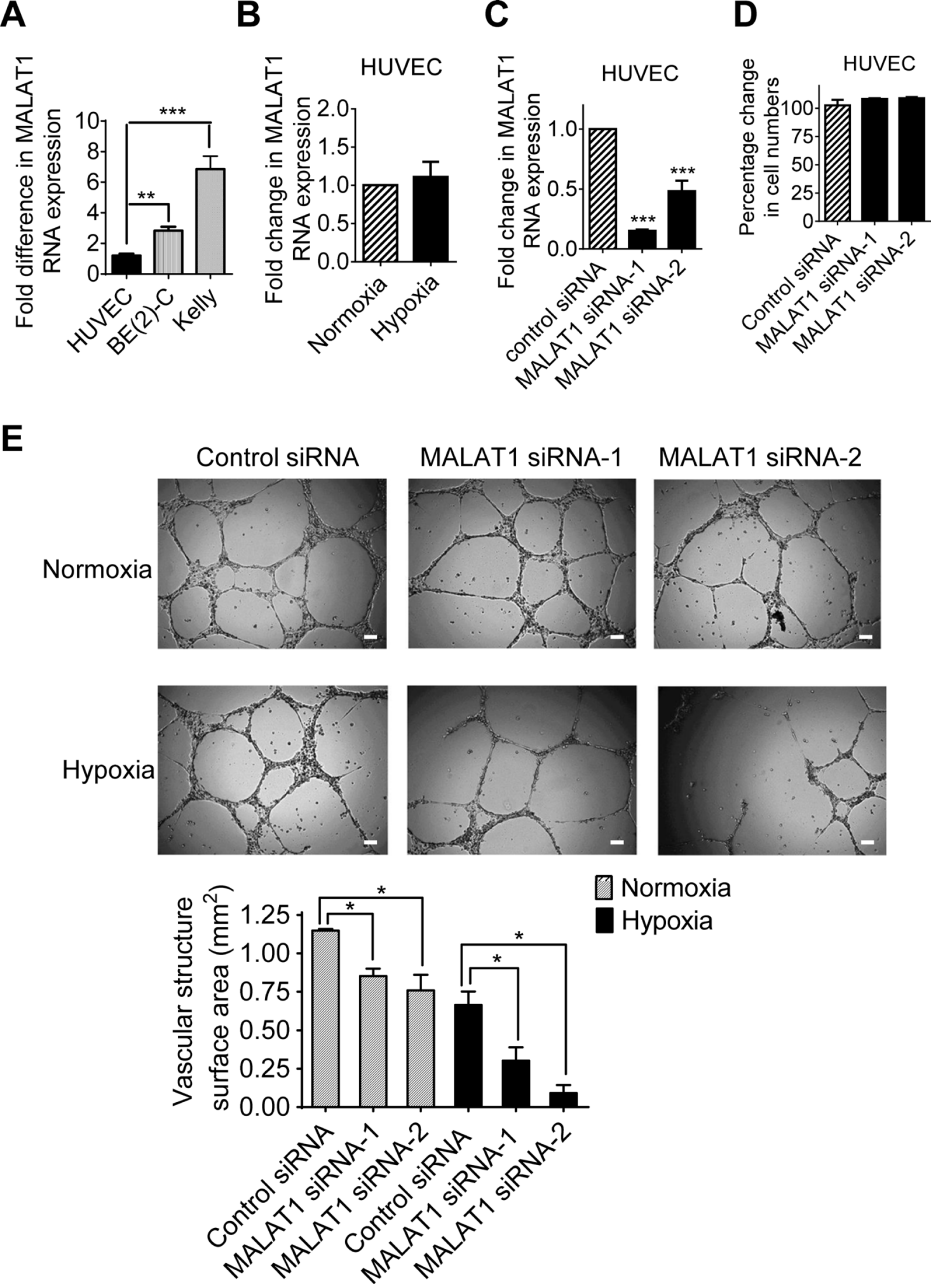
MALAT1 expression in endothelial cells induces vasculature formation **A.** RNA was extracted from BE(2)-C and Kelly neuroblastoma and HUVEC cells under hypoxic conditions, followed by RT-PCR analysis of MALAT1 gene expression. **B.** HUVEC cells were cultured under normoxic or hypoxic conditions for 48 hours, followed by RT-PCR analysis of MALAT1 gene expression. **C.** HUVEC cells were transfected with control siRNA, MALAT1 siRNA-1 or MALAT1 siRNA-2 for 48 hours under hypoxic conditions, followed by RT-PCR analysis of MALAT1 gene expression. **D.** HUVEC cells were transfected with control siRNA, MALAT1 siRNA-1 or MALAT1 siRNA-2 for 72 hours under hypoxic conditions, followed by Alamar blue assays. Relative numbers of cells were expressed as a percentage change compared with samples transfected with control siRNA. **E.** For vasculature formation assays, HUVEC cells were transfected with control siRNA, MALAT1 siRNA-1 or MALAT1 siRNA-2 and cultured under normoxia or hypoxia conditions for 72 hours. Equal numbers of transfected HUVEC cells were then plated into matrigel coated 24-well plates for 8 hours. Photographs of vascular structures were taken using a 5 × objective. Vasculature formation was evaluated by measuring the total surface area of capillary tubes formed in at least 10 randomly selected fields per well. Scale bars represented 100 μm. Error bars represented standard error. * and *** indicated *p* < 0.05 and 0.001 respectively.

We next examined whether MALAT1 expression in endothelial cells stimulates vasculature formation. As shown in Figure 3A and Figure 3B, MALAT1 gene expression was significantly lower in HUVEC cells than in BE(2)-C and Kelly neuroblastoma cells (Figure 3A), and MALAT1 gene expression was not altered in HUVEC cells under hypoxic conditions, compared with those under normoxic conditions (Figure 3B). While transfection with MALAT1 siRNAs significantly reduced MALAT1 gene expression (Figure 3C), Alamar blue assays showed that knocking-down MALAT1 had no effect on HUVEC cell proliferation (Figure 3D). For vasculature formation assays, HUVEC cells were transfected with control siRNA, MALAT1 siRNA-1 or MALAT1 siRNA-2 for 72 hours under either normoxia or hypoxia. Cells were then detached, and equal numbers of transfected cells were cultured on matrigel for 6 hours. As shown in Figure 3E, hypoxic conditions, compared with normoxic conditions, reduced vasculature formation capacity of HUVEC cells. Importantly, under both normoxic and hypoxic conditions, knocking-down MALAT1 significantly reduced vasculature formation (Figure 3E). The data suggest that MALAT1 expression in endothelial cells induces vasculature formation.

### MALAT1 up-regulates FGF2 gene expression in neuroblastoma cells and FGF2 protein secretion under hypoxic conditions

**Figure 4:**
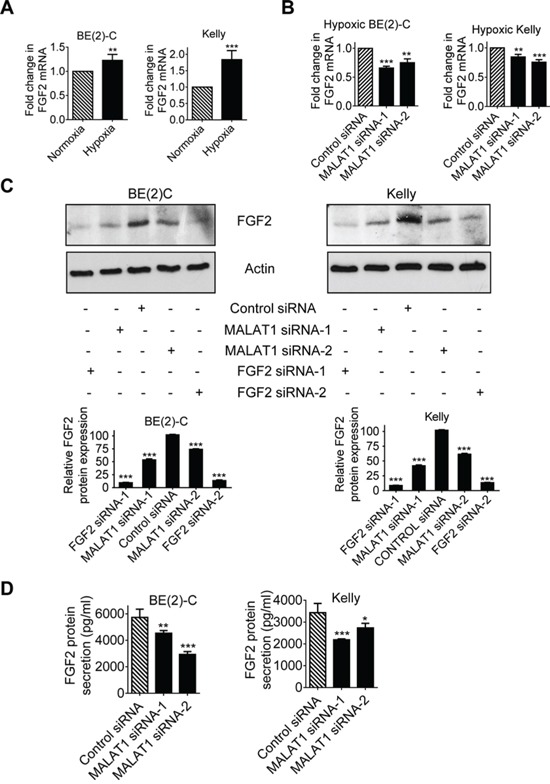
MALAT1 up-regulates FGF2 gene expression in neuroblastoma cells and FGF2 protein secretion under hypoxia conditions **A.** BE(2)-C and Kelly cells were cultured under normoxic or hypoxic conditions for 48 hours. FGF2 gene expression was examined by real-time RT-PCR. **B & C.** BE(2)-C and Kelly cells were transfected with control siRNA, MALAT1 siRNA-1 or MALAT1 siRNA-2 under hypoxic conditions for 48 hours, followed by RT-PCR **(B)** and immunoblot **(C)** analyses of FGF2 mRNA and protein expression respectively. FGF2 protein expression in the immunoblot data was semi-quantified. **D.** BE(2)-C and Kelly cells were transfected with control siRNA, MALAT1 siRNA-1 or MALAT1 siRNA-2 under hypoxic conditions for 72 hours. Cell culture media were collected, and subjected to ELISA analysis of FGF2 protein expression. Error bars represented standard error. *, ** and *** indicated *p* < 0.05, 0.01 and 0.001 respectively.

Since long noncoding RNAs exert biological effects by modulating gene expression [6, 18], we performed differential gene expression studies using Affymetrix microarray in BE(2)-C cells after transfection with control siRNA, MALAT1 siRNA-1 or MALAT1 siRNA-2 for 30 hours. The microarray data showed that one of the genes significantly down-regulated by MALAT1 was FGF2 (Table 1), which is well known to induce angiogenesis [19]. Real-time RT-PCR studies confirmed that hypoxia, compared with normoxia, and immunoblot studies under hypoxia, increased FGF2 gene expression in BE(2)-C and Kelly neuroblastoma cells (Figure 4A), and that knocking-down MALAT1 with siRNAs reduced FGF2 mRNA and protein expression in BE(2)-C and Kelly cells under hypoxic conditions (Figures 4B, 4C).

**Table 1:** The top genes considerably down- or up-regulated by MALAT1 siRNAs in neuroblastoma cells under hypoxic conditions

Probe ID	Gene symbol	Fold change
16983652	CDH6	−1.81
16705011	DKK1	−1.82
16970404	FGF2	−1.82
16845440	SOST	−1.88
16701630	OR2T35	−1.92
17117110	CD24	−1.93
16921664	CXADR	−1.93
17011893	TPI1P3	−1.98
16658926	TARDBP	−1.98
17111008	NUDT11	−1.98
16684192	SNORD99	−2.00
16793678	HIF1A-AS2	−2.00
16860418	CCNE1	−2.00
16778918	KRTAP21-1	−2.03
17096285	CTSL2	−2.03
16809596	RAB27A	−2.04
17109820	KLHL15	−2.08
16868986	ELAVL3	−2.09
17046422	ZNF735	−2.10
16889627	SNORD70	−2.14
16726891	MALAT1	−2.20
16804031	SCARNA15	−2.23
16705715	EIF4EBP2	−2.34
17100667	RFC1	−2.50
17015324	NRN1	−2.84
16819252	MT1F	−2.86
17114478	MMGT1	−3.31
17013657	ULBP1	−3.57
16826738	MT1G	−4.15
16815735	ABAT	2.59
16797415	IGHA1	2.31
16679546	DESI2	2.07
16834749	RUNDC3A	2.06
16809687	NEDD4	1.96
16872254	DYRK1B	1.94
17102418	FAM47B	1.91
16792268	TRAPPC6B	1.86
16793052	GNPNAT1	1.85
16666392	FAM73A	1.84
17118852	TAF1D	1.82

BE(2)-C cells were transfected with control siRNA, MALAT1 siRNA-1 or MALAT1 siRNA-2 for 30 hours under hypoxic conditions. RNA was extracted from the cells, and subjected to differential gene expression study with Affymetrix microarray.

Pro-angiogenic factors are secreted into extracellular fluid, and thereafter stimulate endothelial cell function. We therefore examined whether MALAT1 regulated FGF2 protein secretion into cell culture media. Enzyme-linked immunosorbent assay (ELISA) was performed with conditioned cell culture media from BE(2)-C and Kelly cells 72 hours after transfection with control siRNA, MALAT1 siRNA-1 or MALAT1 siRNA-2 under hypoxic conditions. Knocking-down MALAT1 expression with siRNAs consistently reduced the levels of FGF2 protein in cell culture media (Figure 4D). Taken together, the data suggest that MALAT1 up-regulates FGF2 gene expression in neuroblastoma cells and FGF2 protein secretion into extracellular fluid under hypoxic conditions.

### MALAT1-mediated FGF2 protein secretion from neuroblastoma cells induces vasculature formation

**Figure 5:**
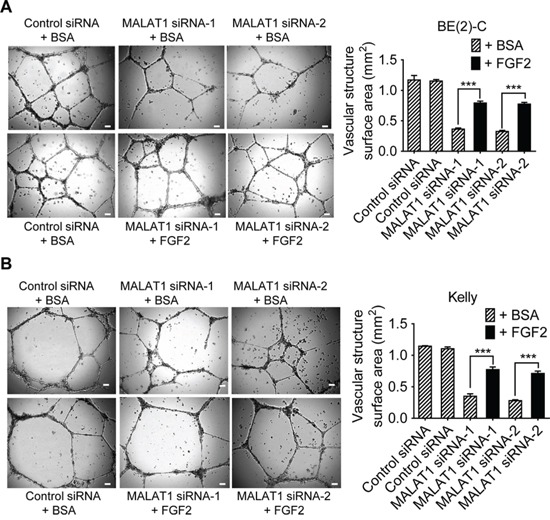
MALAT1-mediated FGF2 protein secretion from neuroblastoma cells induces vasculature formation **A & B.** BE(2)-C **(A)** and Kelly **(B)** neuroblastoma cells were transfected with control siRNA, MALAT1 siRNA-1 or MALAT1 siRNA-2 for 72 hours under hypoxic condition. Cell culture media were collected and divided into two groups. BSA or recombinant FGF2 protein was added into the conditioned cell culture media, according to the ELISA data (Figure 4D), so that the levels of FGF2 protein in MALAT1 siRNA-transfected samples were the same as in control siRNA-transfected samples. The conditioned cell culture media with BSA or recombinant FGF2 protein were then added to HUVEC cells in 24-well plates coated with matrigel for 8 hours at 37°C. Photographs of vascular structures were taken using a 5 × objective. Vasculature formation was evaluated by measuring the total surface area of capillary tubes formed in at least 10 randomly selected fields per well. Scale bars represented 100 μm. Error bars represented standard error. *** indicated *p* < 0.001.

We next examined whether increased secretion of FGF2 protein from neuroblastoma cells was responsible for MALAT1-mediated endothelial cell function. BE(2)-C and Kelly cells were transfected with control siRNA, MALAT1 siRNA-1 or MALAT1 siRNA-2 for 72 hours under hypoxic condition. Conditioned cell culture media were collected and divided into two sub-groups. For one sub-group, recombinant FGF2 protein was added into conditioned cell culture media from cells transfected with MALAT1 siRNAs and equal amount of bovine serum albumin (BSA) was added into conditioned cell culture media from cells transfected with control siRNA, according to the ELISA data (Figure 4D), so that the levels of FGF2 protein in MALAT1 siRNA-transfected samples were the same as in control siRNA-transfected samples. For the other sub-group, BSA was added into conditioned cell culture media from cells transfected with control siRNA or MALAT1 siRNAs for comparison. The conditioned cell culture media were then added to HUVEC cells (Figures 5A, 5B). Vasculature formation assays showed that knocking-down MALAT1 expression in neuroblastoma cells reduced vasculature formation of HUVEC cells, and that addition of FGF2 protein in the conditioned cell culture media largely blocked the effect of MALAT1 siRNAs on vasculature formation.

**Figure 6:**
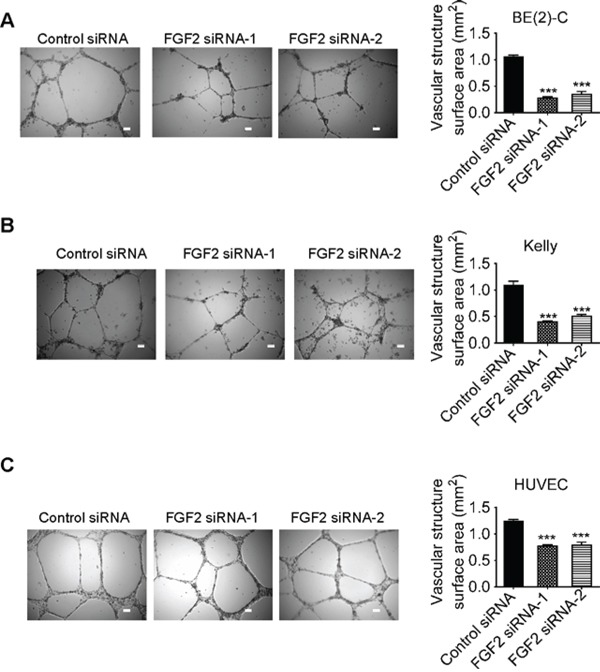
Knocking-down FGF2 expression in neuroblastoma or endothelial cells reduces vasculature formation **A & B.** BE(2)-C **(A)** and Kelly **(B)** neuroblastoma cells were transfected with control siRNA, FGF2 siRNA-1 or FGF2 siRNA-2 for 72 hours under hypoxic conditions. Cell culture media were collected and added to HUVEC cells in 24-well plates coated with matrigel for 8 hours at 37°C. **C.** HUVEC cells were transfected with control siRNA, FGF2 siRNA-1 or FGF2 siRNA-2 for 72 hours. Equal numbers of transfected HUVEC cells were then plated into matrigel coated 24-well plates for 8 hours at 37°C. Photographs of vascular structures were taken using a 5 × objective. Vasculature formation was evaluated by measuring the total surface area of capillary tubes formed in at least 10 randomly selected fields per well. Scale bars represented 100 μm. Error bars represented standard error. *** indicated *p* < 0.001.

We finally transfected BE(2)-C and Kelly cells with control siRNA, FGF2 siRNA-1 or FGF2 siRNA-2 under hypoxic conditions for 72 hours. Conditioned cell culture media were collected and added into HUVEC cells. Vasculature formation assays showed that knocking-down FGF2 gene expression in neuroblastoma cells reduced HUVEC cell vasculature formation (Figures 6A, 6B). In addition, knocking-down FGF2 expression in HUVEC cells also reduced vasculature formation (Figure 6C).

Taken together, the data suggest that MALAT1-mediated FGF2 protein secretion from neuroblastoma cells induces vasculature formation.

## DISCUSSION

During tumor progression, hypoxia develops due to rapid tumor growth and consequent oxygen deficiency. The profound ability of tumors to activate an angiogenic switch under hypoxic conditions and to thereafter trigger angiogenesis is considered a hallmark of malignancies [3]. While neuroblastoma is well known to be characterized by extensive angiogenesis [2], it is not clear which factors are important for neuroblastoma-driven angiogenesis.

The long noncoding RNA MALAT1 is highly expressed in tumor cells of various organ origins, and is expressed at higher levels in metastatic, compared with primary, tumor tissues [11, 12, 17]. We have previously shown that MALAT1 gene expression is up-regulated by the histone demethylase JMJD1A, which is over-expressed in neuroblastoma cells due to N-Myc oncogene amplification, and that MALAT1 induces neuroblastoma cell migration and invasion [16]. In this study, we have found that MALAT1 expression is significantly up-regulated in neuroblastoma cells under hypoxic conditions, compared with normoxic conditions. As HIF1α is well-known to be the master factor in hypoxia-driven gene transcription, it is likely that up-regulation of MALAT1 under hypoxic conditions is due to HIF1α. While knocking-down MALAT1 does not have an effect on neuroblastoma cell proliferation, conditioned media from neuroblastoma cells transfected with MALAT1 siRNAs, compared with conditioned media from control siRNA-transfected neuroblastoma cells, induces less endothelial cell migration, invasion and vasculature formation under hypoxic conditions. The data suggest that up-regulation of MALAT1 by hypoxia contributes to neuroblastoma-driven angiogenesis.

Genetic ablation of MALAT1 in endothelial cells has been reported to reduce neonatal retina vascularization [20]. In the current study, our data show that knocking down MALAT1 gene expression in endothelial cells considerably reduces vasculature formation under both hypoxic and normoxic conditions. However, knocking-down MALAT1 gene expression in endothelial cells has no effect on endothelial cell proliferation. Our findings therefore suggest that MALAT1 expression plays a dual role in tumor-driven angiogenesis, indirectly through MALAT1-mediated crosstalk between tumor cells and endothelial cells and directly through endothelial cells, and that targeting this long noncoding RNA may be an effective strategy for blocking both tumor metastasis and angiogenesis in neuroblastoma. While no small molecule inhibitors specifically targeting MALAT1 are currently available, resveratrol has been shown to down-regulate MALAT1, resulting in decreased Wnt/beta-catenin signalling, reduced cancer cell invasion and metastasis [21]. Whether resveratrol can be used to block MALAT1 expression, tumor metastasis and angiogenesis *in vivo* needs further investigation.

lncRNAs are well known to exert biological effects by regulating chromatin status to alter gene transcription [6, 7] and by acting as a competing endogenous RNA to increase target mRNA stability [22]. In this study, our microarray differential gene expression study has identified several genes, including FGF2, significantly regulated by MALAT1 in neuroblastoma cells under hypoxic conditions. While none of the other MALAT1 target genes identified by the microarray data is known to regulate endothelial cell function, FGF2 has been well-documented to regulate endothelial cell migration and vasculature formation via activation of Fibroblast Growth Factor Receptor, and is one of the most important pro-angiogenic factors [23]. FGFR2 signaling also induces chemoresistance in neuroblastoma cells through protein kinase C-δ-dependent induction of BCL2 expression [24]. Consistently, treatment with FGF2 vaccine results in abrogation of angiogenesis and tumor development [25].

In this study, RT-PCR and immunoblot analyses confirm that MALAT1 siRNAs reduce FGF2 mRNA and protein expression, and ELISA assays confirm that knocking-down MALAT1 expression in neuroblastoma cells reduces FGF2 protein secretion into extracellular fluid. Chromatin immunoprecipitation assays show that knocking-down MALAT1 does not have an effect on the presence of tri-methylated histone H3 lysine 4 (data not shown), a marker for active gene transcription, suggesting that MALAT1 up-regulates FGF2 mRNA through a post-transcriptional mechanism, possibly through acting as a competing endogenous RNA and increasing FGF2 mRNA stability. Importantly, we have further demonstrated that addition of recombinant FGF2 protein into conditioned cell culture media from neuroblastoma cells transfected with MALAT1 siRNAs largely blocks MALAT1 siRNA-mediated reduction in vasculature formation of endothelial cells. In addition, knocking-down FGF2 expression in neuroblastoma cells or endothelial cells both reduces vasculature formation. Taken together, our data suggest that MALAT1 gene expression in human neuroblastoma cells induces vasculature formation of endothelial cells mainly through up-regulating FGF2 gene expression.

In summary, MALAT1 gene expression is up-regulated in neuroblastoma cells following hypoxia. While up-regulation of MALAT1 does not promote neuroblastoma cell proliferation, it up-regulates FGF2 mRNA and protein expression in neuroblastoma cells and increases FGF2 protein secretion into extracellular fluid, leading to endothelial cell migration, invasion and vasculature formation. MALAT1 gene up-regulation under hypoxic condition, therefore, plays an important role in tumor-driven angiogenesis and represents a potentially valuable therapeutic target for the treatment of this disease.

## MATERIALS AND METHODS

### Cancer cell line culture

Human BE(2)-C and Kelly neuroblastoma cells were cultured in Dulbecco's modified Eagle's and RPMI 1640 media, respectively, supplemented with 10% fetal calf serum. BE(2)-C cells were obtained from American Type Culture Collection, and Kelly cells from European Collection of Cell Cultures. The identity of the cell lines was verified by small tandem repeat profiling conducted at Garvan Institute and Cellbank Australia (Sydney, New South Wales, Australia). Cells were cultured under normoxic (with gas mixture containing 21% O_2_, 5% CO_2_ and 74% nitrogen) or hypoxic (with gas mixture containing 1% O_2_, 5% CO_2_ and 94% nitrogen) condition.

### Endothelial cell culture

HUVEC cells were purchased from American Type Culture Collection, and maintained in Medium 199 supplemented with 20% fetal bovine serum, 5% human serum (Sigma, St Louis, MO), 10 U/ml heparin (Pharmacia & Upjohn, Peapack, NJ), 5 ng/ml FGF2 (Sigma) and 20 μg/mL Endothelial Cell Growth Factor (Roche, Mannheim, Germany). Only passage five HUVEC cells were used in the experiments.

### siRNA and plasmid transfection

Cells were transfected with siRNAs from Qiagen (Qiagen, Hamburg, Germany) or Ambion (Ambion, Austin, TX, USA) using Lipofectamine 2000 (Invitrogen, Carlsbad, CA) reagent as previously described [8, 26]. The target sequences for MALAT1 siRNA-1 and MALAT1 siRNA-2 were CACAGGGAAAGCGAGUGGUUGGU and GACAGGUAUCUCUUCGUUA, and those for FGF2 siRNA-1 and FGF2 siRNA-2 TAGCACTAGTCTTAAATTGTA and AACAATATTA GTCGTATCCAA.

### RT-PCR and immunoblot analyses

Gene expression in tumor and HUVEC cells was examined by quantitative real-time RT-PCR as described previously [8, 26]. Sequences of specific primers used for RT-PCR were as the following: 5′-GAATTGCGTCATTTAAAGCCTAG-3′ and 5′-GTTTCATCCTACCACTCCCAATT-3′ for MALAT1; 5′-CCGTTACCTGGCTATGAAGG-3′ and 5′-ACTGCCCAGTTCGTTTCAGT-3′ for FGF2; and 5′-AGGCCAACCGCGAGAAG-3′ and 5′-ACAGCCTGGATAGCAACGTACA-3′ for β-actin. For the analysis of protein expression by immunoblot, cells were lysed, protein extracted and separated by gel electrophoresis. After western transfer, membranes were probed with rabbit anti-FGF2 antibody (1:250) (Santa Cruz Biotech, Santa Cruz, CA), followed by horseradish peroxidase-conjugated anti-rabbit (1:10,000) antiserum (Santa Cruz Biotech, Santa Cruz, CA). Protein bands were visualized with SuperSignal (Pierce, Rockford, IL). The membranes were lastly re-probed with a mouse anti-actin antibody (Sigma) as loading controls.

### FGF2 protein ELISA

BE(2)-C and Kelly cells were transfected with control siRNA, MALAT1 siRNA-1 or MALAT1 siRNA-2 under hypoxic condition for 72 hours. Cell culture media was collected and subjected to measurement of FGF2 protein with the sandwich ELISA kit (R&D Systems, Minneapolis, MN), as described by the manufacturer. Plates were read with a microplate reader at 450 nm with background correction at 570 nm.

### Affymetrix gene array study

BE(2)-C neuroblastoma cells were transfected with scrambled control siRNA, MALAT1 siRNA-1 or MALAT1 siRNA-2. Thirty hours after transfection, RNA was extracted from the cells with RNeasy mini kit (Qiagen). Differential gene expression was examined with Affymetrix HuGene-1_0-st-v1 Arrays (Affymetrix, Santa Clara, CA), according to the manufacturer's instructions. The LimmaGP module (Gene Pattern, Broad Institute, Cambridge, MA) was used to identify genes differentially expressed between samples.

### Cell proliferation assays

Cell proliferation was examined with Alamar blue assays [27]. Briefly, cells were plated into 96 well plates, transfected with various siRNAs and grown under hypoxic conditions. Seventy-two hours later, cells were incubated with Alamar blue (Invitrogen) for 6 hours, and plates were then read on a micro-plate reader at 570/595 nm. Results were calculated according to the optical density absorbance units and expressed as percentage changes in cell numbers.

### Transwell HUVEC cell migration assays

Transwell chemotaxis cell migration assays were performed as previously described [28]. Briefly, the underside of 8 μm transparent polyethylene terephthalate membrane inserts (BD Biosciences, San Jose, CA) was pre-coated with 0.1% gelatin for 1 hour. The cells were pre-labeled *in situ* with 10 μM Cell Tracker Green CMFDA (Invitrogen) in serum-free medium for 30 minutes and 100,000 cells were then seeded onto the insert in serum-free assay medium containing 0.5% BSA. Assay medium supplemented with 5% fetal calf serum was then added to the bottom of the insert and used as chemoattractant. A negative control was included in each experiment by adding serum-free medium to the bottom of the insert. The plates were incubated for 6 hours at 37°C and 5% CO_2_. Excess cells on the upper side of the insert were then gently swabbed off with a cotton tip and migrated cells at the underside of the insert were measured with a Victor 3 plate reader (Perkin-Elmer, Glen Waverley, Australia) at 492/517 (Absorbance/Emission). All readings were normalized to the negative control (serum-free medium).

### HUVEC cell invasion assays

HUVEC cell invasion assays were carried out using BD BioCoat Growth Factor Reduced Matrigel Invasion Chambers according to the manufacturer's guidelines (BD Biosciences) and as we described previously [29]. Neuroblastoma cells were transfected with control siRNA, MALAT1 siRNA-1 or MALAT1 siRNA-2, followed by serum starvation for 72 hours and collection of conditioned cell culture media. The neuroblastoma cell conditioned media were added into the lower part of cell invasion chambers. HUVEC cells were plated into the upper part of the invasion chambers and allowed to invade through membranes coated with matrigel towards the conditioned cell culture media overnight for 18 hours at 37°C. Cells invaded through the membrane were fixed with 100% methanol, stained with May-Grunwald and Giemsa (Sigma-Aldrich), visualized using the Olympus BH-2 microscope and quantified.

### HUVEC cell vasculature formation assays

Matrigel (BD Biosciences) was diluted 1:1 in M199 medium supplemented with 0.1% fetal calf serum, 10U/ml heparin, 5 ng/ml FGF2, 20 μg/ ml Endothelial Cell Growth Factor and 29.2 mg/ml glutamine. The Matrigel solution was dispensed into 24-well plates and incubated for 1 hour at 37°C. HUVEC cells were overlaid onto the Matrigel at 7.5 × 10^4^ cells per well and incubated at 37°C for 8 hours. Vascular structures (enclosed polygons) were photographed using the 5 × objective of an Axiovert 200M fluorescent microscope coupled to an AxioCamMR3 camera (Carl Zeiss, Munich, Germany). Ten fields of views were photographed for each condition and the numbers of vascularature-like structures were quantified using the Zeiss AxioVision 4.7 Image Analysis software.

### Statistical analysis

All experiments were repeated for at least 3 times for statistical analysis. All data for statistical analysis were calculated as mean ± standard error. Differences were analyzed for significance using ANOVA among groups or unpaired t-test for two groups. A probability value of 0.05 or less was considered significant.
